# miR-942 promotes cancer stem cell-like traits in esophageal squamous cell carcinoma through activation of Wnt/β-catenin signalling pathway

**DOI:** 10.18632/oncotarget.3696

**Published:** 2015-03-30

**Authors:** Chunlei Ge, Shikai Wu, Weiwei Wang, Zhimin Liu, Jianhua Zhang, Zhenyu Wang, Ruilei Li, Zhiwei Zhang, Zhen Li, Suwei Dong, Ying Wang, Yuanbo Xue, Jinyan Yang, Qinghua Tan, Ziping Wang, Xin Song

**Affiliations:** ^1^ Department of Cancer Biotherapy Center, The Third Affiliated Hospital of Kunming Medical University (Tumor Hospital of Yunnan Province), Kunming, Yunnan, People's Republic of China; ^2^ Department of Radiation Oncology, Affiliated Hospital of Academy of Military Medical Sciences, Beijing, People's Republic of China; ^3^ Department of Thoracic Surgery, The Third Affiliated Hospital of Kunming Medical University (Tumor Hospital of Yunnan Province), Kunming, Yunnan, People's Republic of China; ^4^ Department of Biomedical Engineering Research Center, Kunming Medical University, Kunming, Yunnan, People's Republic of China; ^5^ Department of Medical Oncology, Cancer Institute and Hospital, Chinese Academy of Medical Sciences (CAMS), Beijing, People's Republic of China

**Keywords:** miR-942, cancer stem cells, Wnt/β-catenin signalling pathway, esophageal squamous cell carcinoma

## Abstract

The Wnt/β-catenin signalling pathway is known to play a vital role in the maintenance of cancer stem cells (CSCs), which are reported to be the origine of malignant cancers, and result in poor prognosis of multiple kinds of cancer. Therefore, it is of great importance to illuminate the mechanism by which the Wnt/β-catenin pathway regulates the cancer stem cell-like traits in cancers. Here, we report that miR-942 is significantly upregulated in esophageal squamous cell carcinoma (ESCC), and miR-942 levels are associated with poor prognosis in ESCC patients. Overexpression of miR-942 promotes, whereas inhibition of miR-942 decreases, the tumor sphere formation, the CD90^+^ subpopulation cells and the expression of pluripotency associated markers. Moreover, *in vivo* assay shows that miR-942 overexpressing cells form larger tumors and display higher tumourigenesis. Furthermore, we demonstrate that miR-942 upregulates the Wnt/β-catenin signaling activity *via* directly targeting sFRP4, GSK3β and TLE1, which are multiple level negative regulators of the Wnt/β-catenin signaling cascade. In addition, our results indicate that c-myc directly binds to the miR-942 promoter and promotes its expression. Taken together, our findings establish an oncogenic role of miR-942 in ESCC and indicate that miR-942 might be an effective therapeutic target for ESCC.

## INTRODUCTION

Cancer stem cells (CSCs), a small population of cancer cells that possess the ability to self-renew and differentiate, are generally responsible for cancer initiation and poor prognosis [1-3]. It has been reported that CSCs exhibit both stem cell-like traits and cancer properties. Only a small portion of CSCs, or even a single CSC, can form a tumour [4]. Meanwhile, CSCs exhibit greater resistance to cancer treatment than cancer cells, such as chemotherapy and radiotherapy [5, 6]. Several studies have reported that CSCs play a vital role in esophageal squamous cell carcinoma (ESCC) progression. For example, Forghanifard et al. found that SALL4 and SOX2, which are stemness state transcriptional factors involving in maintenance of pluripotency and self-renewal, played vital role in ESCC progression [7]. Zhao and colleagues have demonstrated the stem cell-like side populations are a source of chemo-resistance and metastasis in esophageal cancer [8]. Therefore, targeting CSCs could be an effective treatment for ESCC therapy. And it is vital to understand the molecular mechanisms of which control CSCs in ESCC.

The Wnt/β-catenin signalling pathway, a key molecular pathway controlling the function of stem cells, has been shown to play an important role in CSCs [9-11]. The canonical Wnt/β-catenin signaling is stimulated by the secreted Wnt ligands, which bind to the Frizzled (FZD) family receptors and LRP5/LRP6 co-receptor to trigger the β-catenin signalling cascade. Receptors activation leads to the phosphorylation of Dishevelled (Dvl), and consequently release β-catenin from the “destruction complex” - Axin, adenomatous polyposis coli (APC), casein kinase 1α (CK1α), and glycogen synthase kinase 3β (GSK3β). Thereby, β-catenin translocates to the nucleus and binds to the N-terminal of the TCF/LEF transcriptional factor, thus activating downstream target genes [12-14]. The Wnt/β-catenin pathway has been shown to be constitutively activated in various types of cancer, leading to cell reprogramming and generation of a stem-like phenotype [15, 16]. Numerous studies have shown that Wnt/β-catenin signalling is hyperactivated in multiple cancers [12, 17]. In colon cancer, inappropriate permanent activation of the Wnt cascade always due to the loss of APC allele, Axin mutation or activating β-catenin point mutation [17, 18]. Tanaka et al. have reported that a novel member of the human frizzled (FzD) gene family FzE3 was expressed only in esophageal cancer tissues, but not in adjacent normal tissues [19].

On the other hand, dysregulation of negative regulators could also contribute to constitutive activation of the Wnt/β-catenin signalling in cancer. These negative regulators are involved in three layers in Wnt/β-catenin signalling: extracellular secreted Wnt inhibitors, such as the secreted Frizzled-related proteins (sFRPs), which serve as Wnt antagonists and inhibit Wnt binding to Frizzled receptors [20-22]; intracellular canonical Wnt inhibitors- Axin, adenomatous polyposis coli (APC), casein kinase 1α (CK1α), and glycogen synthase kinase 3β (GSK3β), which form the destruction complex and prevent β-catenin from translocating into the cell nucleus [23]; and nuclear transcriptional suppressors, such as TLE1, NLK and CtBP2, which inhibit the transcriptional activity of LEF/TCF [24-26]. However, how these negative regulators of the Wnt/β-catenin signalling pathway are concomitantly deregulated in cancer is largely unclear.

In the present study, we found that the microRNA miR-942 was markedly overexpressed in ESCC and promoted the stem cell-like traits by inhibition of three negative regulators of Wnt/β-catenin pathway, including sFRP4, GSK3β and TLE1. Therefore, our results suggest that miR-942 might be a potential therapeutic target for ESCC.

## RESULTS

### miR-942 overexpression correlates with ESCC progression

**Figure 1:**
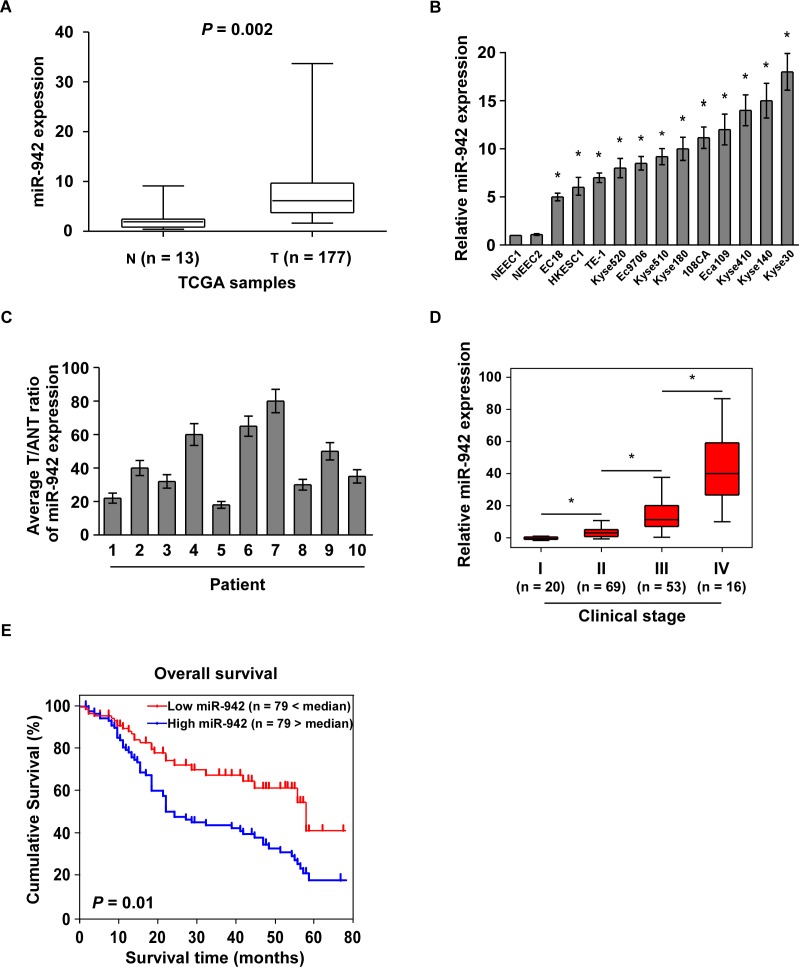
Overexpression of miR-942 is positively associated with ESCC prognosis (**A**) Expression profiling of miR-942 from the Cancer Genome Atlas (TCGA) datasets in primary esophageal tumours (T, n=177) and the adjacent normal tissues (ANT, n=13) (*P* = 0.002). (**B-C**) Real-time PCR analysis of miR-942 expression in 2 NEECs and 12 ESCC cell lines (**B**), as well as 10 paired ESCC samples (T) and adjacent normal tissues (ANT). Transcript levels were normalized by to *U6* expression. Each bar represents the mean ± SD of three independent experiments. * *P* < 0.05. (**D**) Correlation of miR-942 expression in WHO grading of ESCC assessed by real-time PCR. Transcript levels were normalized by *U6* expression. The boundaries of the boxes represent the lower and upper quartiles; lines within boxes and whiskers denote median and extremum, respectively. * *P* < 0.05 (**E**) Kaplan-Meier curves of ESCC patients with low- versus high-expression of miR-942 (*n* = 158; *P* <0.001, log-rank test).

By analysis the Cancer Genome Atlas (TCGA) microarray data set consisting of 177 primary esophageal cancer tissues and 13 normal esophageal tissues, miR-942 was significantly upregulated in tumour tissues compared to normal tissues (Fig. 1A and Supplementary Fig. 1). Furthermore, real-time PCR analysis showed that miR-942 was ubiquitously overexpressed in 10 ESCC samples compared with the paired normal tissues and in all 12 ESCC cell lines compared with 2 NEECs (Fig. 1B and 1C). Collectively, these findings suggested that miR-942 expression is significantly increased in ESCC.

The observed upregulation of miR-942 prompted us to further investigate the clinical relevance of miR-942 in ESCC progression. We therefore examined miR-942 expression in a cohort of 158 archived human ESCC specimens (Supplementary Table 1). Statistical analysis revealed that miR-942 expression was positively correlated with clinical stage (*P* < 0.001), tumour–node–metastasis (TNM) classification (T: *P* = 0.005; N: *P* < 0.001; M: *P* = 0.004), and histologic differentiation (*P* = 0.045) in patients with ESCC (Fig. 1D and Supplementary Table 2). Importantly, patients with higher miR-942 expression had a shorter survival time, whereas patients with lower miR-942 expression had a longer survival time (*P* = 0.01; Fig. 1E). Moreover, Univariate and multivariate analyses indicated that miR-942 expression and clinical stage were independent prognostic factors in ESCC (Supplementary Table 3). Taken together, these results indicate a possible link between miR-942 overexpression and human ESCC progression.

### Upregulation of miR-942 promotes cancer stem cell-like traits in ESCC

**Figure 2:**
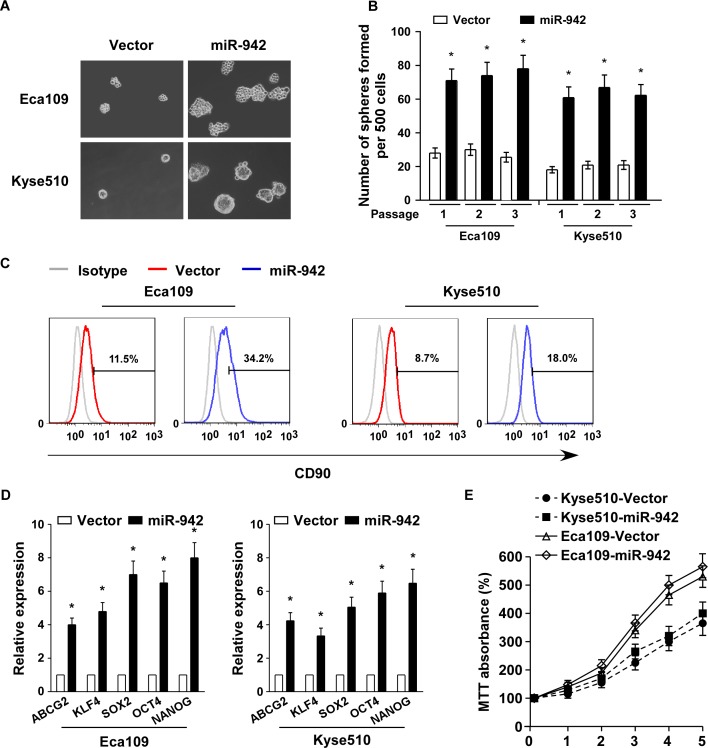
miR-942 overexpression promotes cancer stem-like traits in ESCC (**A-B**) Representative micrographs (left) and quantification (right) of tumourspheres formation in miR-942-overexpression or vector cells. Scale bar, 100 μm. (**C**) Effects of miR-942-overexpression cells on the distribution of CD90^+^ cells using flow cytometric analysis. * *P* < 0.05 (**D**) Real-time PCR analysis of the mRNA expression of pluripotency-associated markers in the indicated cells. Transcript levels were normalized to GAPDH expression. Error bars represent mean ± SD from three independent experiments. *P < 0.05 (**E**) MTT assay revealed that miR-942 overexpression is only slightly quick in Eca109 and Kyse510 stable cell lines compare to the vector control cells at indicated times after seeding.

In attempt to understand the biological effect of miR-942 in ESCC progression, miR-942 was stably transduced into the Eca109 and Kyse510 ESCC cell lines to generate Eca109/miR-942 and Kyse510/miR-942 cell lines (Supplementary Fig. 2A). A tumour sphere formation assay showed that miR-942-transduced cells formed more and larger spheres than vector-tranduced cells (Fig. 2A and 2B). Additionally, CD90 positive cells, which were well-known esophageal CSC marker, were dramatically increased in miR-942-transduced cells compared with vector-tranduced cells (Fig. 2C). Furthermore, miR-942 overexpression significantly upregulated the mRNA expression levels of multiple pluripotency factors, including ABCG2, KLF4, SOX2, OCT4, and NANOG (Fig. 2D). However, the proliferative rate of miR-942-transduced Eca109 and Kyse510 cells is only slightly quick compare to the vector control cells (Fig. 2E). Collectively, our results suggest that miR-942 overexpression promotes the stem cell-like traits of ESCC cells.

### miR-942 inhibition suppresses ESCC stem cell-like traits

**Figure 3:**
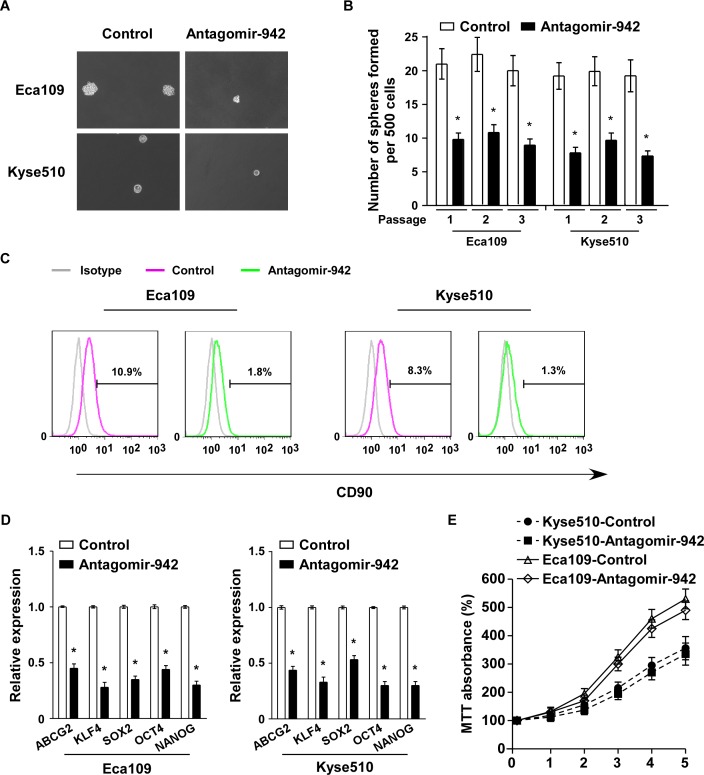
miR-942 inhibition reduces stem cell-like traits in ESCC (**A-B**) Representative micrographs (left) and quantification (right) of tumourspheres formation in antagomir-942 or control cells. Scale bar, 100 μm. (**C**) Effects of antagomir-942 or control cells on the distribution of CD90^+^ cells using flow cytometric analysis. * *P* < 0.05 (**D**) Real-time PCR analysis of the mRNA expression of pluripotency-associated markers in antagomir-942 or control cells. Transcript levels were normalized to GAPDH expression. Error bars represent mean±SD from three independent experiments. * *P* < 0.05. (**E**) MTT assay revealed that inhibition of miR-942 is only slightly suppressed in Eca109 and Kyse510 compare to the control cells at indicated times after seeding.

To examine the role of endogenous miR-942 in ESCC stem cell-like traits, antagomir-942, an antisense-based specific inhibitor against miR-942, was applied as antagonists to silence endogenous miR-942 (Supplementary Fig. 2B). As shown in Fig. 3A and 3B, the tumour sphere formation assay revealed that when miR-942 was inhibited, the cells formed fewer and smaller spheres. Similarly, CD90 population was dramatically decreased in antagomir-942 cells compared with control cells (Fig. 3C). Furthermore, miR-942 inhibition significantly decreased the mRNA expreesion of ABCG2, KLF4, SOX2, OCT4, and NANOG (Fig. 3D). However, inhibition of miR-942 is only slightly suppressed in Eca109 and Kyse510 compare to the control cells (Fig. 3E). Thus, our experiments indicated that endogenous miR-942 might act as a cancer stem cell inducer which promotes ESCC stem cell-like traits.

### Upregulation of miR-942 promotes tumourigenecity of ESCC cells *in vivo*

**Figure 4:**
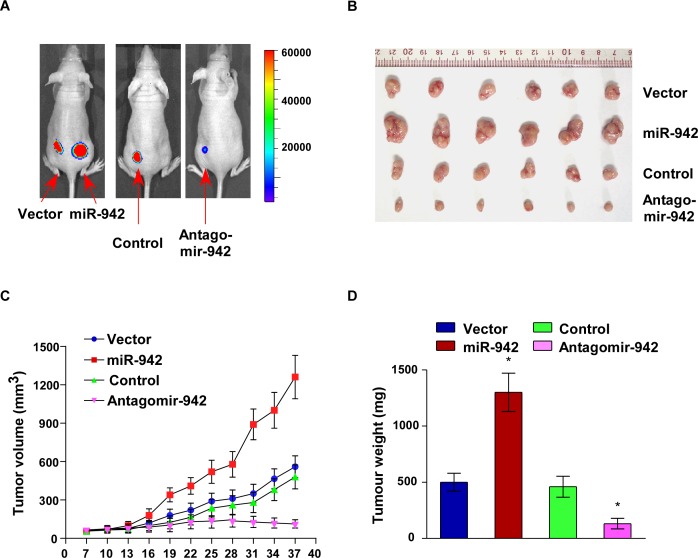
Upregulation of miR-942 increases tumourigenesis of ESCC cells *in vivo* Xenograft model in nude mice. (**A**) Representative images of tumour-bearing mice. (**B**) Images of the tumours from each group. (**C**) Tumour volumes were measured on the indicated days. (**D**) Mean tumour weights. Each bar represents the mean ± SD of three independent experiments. * *P* < 0.05.

The biological effect of miR-942 on ESCC progression was further examined using an *in vivo* tumour model. Eca109/miR-942 or Eca109/vector cells were subcutaneously xenografted into the NOD/SCID mice. As shown in Fig. 4A-4D, the tumours formed by Eca109/miR-942 cells were larger, in both size and weight, than the tumours formed from vector control cells. In contrast, when endogenous expression of miR-942 was inhibited using antagomir-942, the tumours were obviously smaller and lighter than those formed by control cells. The tumours formed by Eca109/miR-942 cells were significantly larger than the vector control tumours, when 1 × 10^4^ or 1 × 10^3^ cells mixed with matrigel were subcutaneously inoculated into the mice. Importantly, only Eca109/miR-942 cells formed tumours when 1 ×10^2^ cells were implanted (Table 1). These results indicated that miR-942 strongly promotes ESCC tumourigenesis *in vivo*.

**Table 1:** Effect of miR-942 on the ability of ESCC cells to form tumours *in vivo*

Cell type	Number of cells inoculated		
	1 × 10^4^	1 × 10^3^	1 × 10^2^
Eca109-vector	6/6	3/6	0/6
Eca109-miR-942	6/6	6/6	5/6
Eca109-NC	6/6	3/6	0/6
Eca109-Antagomir-942	2/6	0/6	0/6

### miR-942 directly targets sFRP4, GSK3β, and TLE1

**Figure 5:**
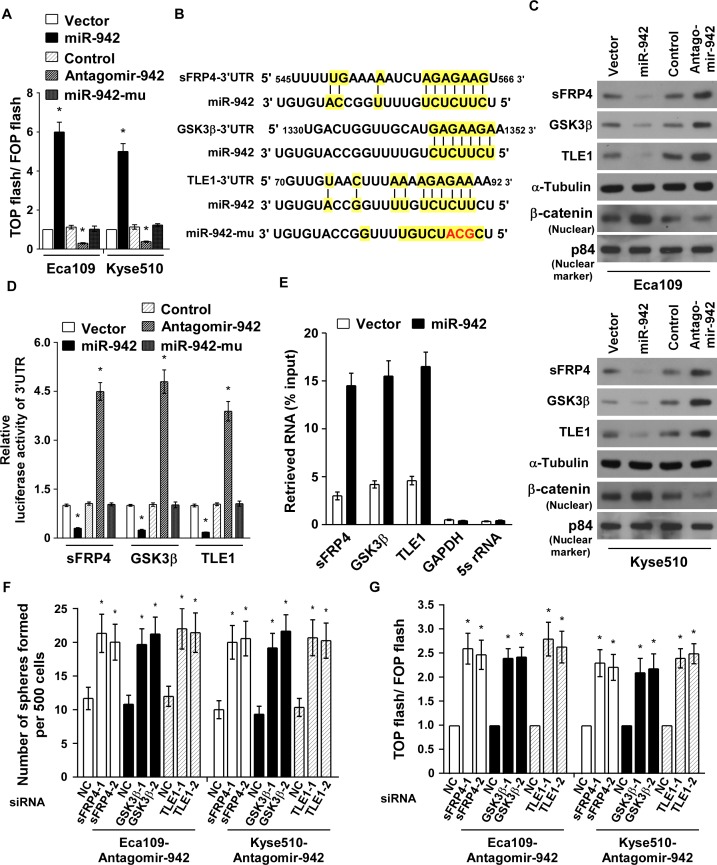
miR-942 directly targets sFRP4, GSK3β, and TLE1 (**A**) Indicated cells transfected with TOP flash/FOP flash and Renilla pRL-TK plasmids were subjected to dual-luciferase assays 48 hours after transfection. Reporter activity detected was normalized by Renilla luciferase activity. (**B**) Predicted miR-942 target sequences in the 3′-UTRs of sFRP4, GSK3β and TLE1 and mutant containing three altered nucleotides in the seed sequence of miR-942 (miR-942-mu). (**C**) Western blotting analysis of sFRP4, GSK3β, TLE1 and nuclear β-catenin in the indicated cells. α-Tubulin served as the loading control. (**D**) Luciferase activity of targets 3′UTR in the indicated cells. (**E**) miRNP immunoprecipitation assay showed associations of miR-942 with sFRP4, GSK3β and TLE1. *GAPDH* and 5s rRNA served as a negative control. (**F**) Quantification of indicated tumoursphere cells with specific siRNA transfection. (**G**) Luciferase activity of TOP flash/FOP flash in the indicated cells. Each bar represents the mean ± SD of three independent experiments. * *P* < 0.05.

As Wnt/β-catenin signalling pathways is the major molecular pathway controlling CSCs and promotes tumour progression [27], we hypothesis miR-942 might regulate Wnt/β-catenin signalling. As expected, miR-942 overexpression markedly increased the luciferase activity of the TOP flash/FOP flash reporter. Conversely, transfection of antagomir-942 reduced the luciferase activity, and mutant miR-942 had no effect (Fig. 5A), indicating that miR-942 activates Wnt/β-catenin signalling. Using publicly available algorithms (TargetScan6.2 and miRanda), we found that sFRP4, GSK3β, and TLE1, the multiple negative regulators of Wnt/β-catenin pathway, might be the potential targets of miR-942 (Fig. 5B). Western blot analysis revealed that the expressions of sFRP4, GSK3β, and TLE1 was drastically decreased in miR-942-transduced cells but pronouncedly elevated in antagomir-942 cells, compared with their corresponding control cells (Fig. 5C). In addition, results of the luciferase reporter activities, which linked to the 3′UTRs of sFRP4, GSK3β, and TLE1, indicated that miR-942 overexpression significantly repressed, whereas inhibition of endogenous miR-942 increased, the luciferase activity of 3′UTRs, and ectopically expressing mutant miR-942 had no inhibitory effect on the 3′UTRs luciferase activity (Fig. 5D). Importantly, results of the miRNP immunoprecipitation assay showed that miR-942 selectively associated with sFRP4, GSK3β, and TLE1, but not GAPDH or 5s rRNA (Fig. 5E). Hence, our results indicate that sFRP4, GSK3β, and TLE1 are the *bona fide* targets of miR-942.

### miR-942 maintains cancer stemness via activating Wnt/β-catenin signalling pathway

Since sFRP4, GSK3β, and TLE1 are important negative regulators of the Wnt/β-catenin pathway, we then examined the effect of miR-942 on the Wnt/β-catenin pathway. Western blot analysis proved that miR-942 overexpression induced, wheras miR-942 inhibition decreased, the nuclear accumulation of β-catenin (Fig. 5C). To explore the functional significance of sFRP4, GSK3β, and TLE1 in stem cell-like traits and β-catenin activation induced by miR-942, we silenced the endogenous sFRP4, GSK3β, and TLE1 using siRNAs in antagomir-942 cells (Supplementary Fig. 3). As shown in Fig. 5F and 5G, individually silencing sFRP4, GSK3β, or TLE1 increased the tumour sphere formation and enhanced the TOP flash/FOP flash activity of antagomir-942 cells. These results demonstated that sFRP4, GSK3β, and TLE1 are important for miR-942-induced stem cell-like traits and indicated that Wnt/β-catenin signalling is a functional mediator for miR-942–induced function in ESCC cell lines.

### miR-942 is upregulated by c-myc and clinically correlated with c-myc and β-catenin

**Figure 6:**
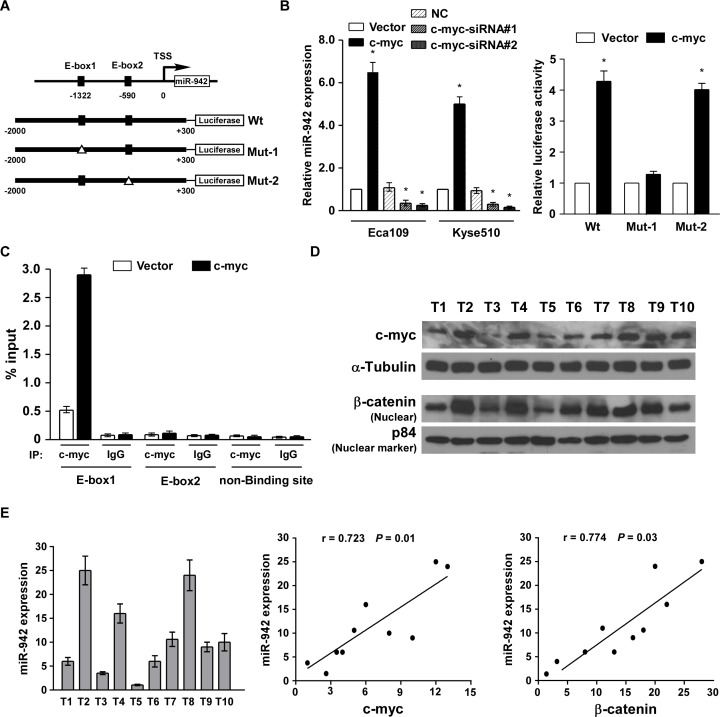
miR-942 is upregulated by c-myc and clinically corelated with c-myc and β-catenin (**A**) Upper: the exact binding sites of c-myc on the human miR-942 promoter. Down: schematic illustration of the cloned fragments of the human miR-942 promoter. E-box1 and E-box2 were mutated to identify which site is essential for c-myc regulation. (**B**) Real-time PCR analysis of miR-942 expression in the cells transfected with c-myc or c-myc siRNA. Transcript levels were normalized by U6 expression. c-myc affects the transactivity of miR-942 promoter with or without E-boxes. (**C**) ChIP-qPCR analyses for the fold enrichment of c-myc at the different promoter regions of human miR-942 in Eca109 cells. (**D**) Analysis of expression of miR-942 with the expression levels of c-myc and nuclear β-catenin in 10 freshly collected human ESCC samples. The ratio of fifth sample (c-myc /α-tubulin, beta-catenin /α-tubulin) was considered as 1.0. Each bar represents the mean ± SD of three independent experiments.

To determine the mechanism of miR-942 upregulation in ESCC, the miR-942 promoter region was analysed using the UCSC genome browser (http://genome.ucsc.edu/), and two binding sites for c-myc (E-box1 and E-box2) were predicted on the promoter of miR-942 (Fig. 6A). Real-time PCR indicated that miR-942 was upregulated in c-myc-elevated cells and was downregulated in c-myc-silenced cells (Fig. 6B). Furthermore, a dual-luciferase reporter assay showed that c-myc obviously activates miR-942 through binding to the first E-box (E-box1) (Fig. 6B). Meanwhile, when mutated the second E-box, no obvious alterations were observed. Moreover, chromatin immunoprecipitation (CHIP) assays showed high binding affinity of endogenous c-myc with the first E-box region (Fig. 6C). Collectively, our results indicated that c-myc directly binds to the promoter of miR-942 and promotes its expression. Finally, using 10 freshly collected clinical ESCC samples, miR-942 expression was shown to be positively correlated with c-myc (r = 0.723, *P* = 0.01), nuclear β-catenin expression (r = 0.774, *P* = 0.03) (Fig. 6D and 6E), further supporting the notion that miR-942 is upregulated by c-myc and promoted stem-cell like traits by activating Wnt/β-catenin signalling in ESCC.

## DISCUSSION

To our knowledge, the present study is the first time to show the central role of miR-942 in promoting ESCC tumourigenesis. Using microRNA screening and real-time PCR assays, we found that miR-942 was strongly overexpressed in ESCC and was positively correlated with poor prognosis in this cancer. Further, miR-942 induced ESCC stem-like traits through directly targeting sFRP4, GSK3β and TLE1, which are important negative regulators of the Wnt/β-catenin pathway. Moreover, c-myc was found to directly bind to miR-942 promoter and upregulates miR-942 expression. Taken together, our results revealed a novel mechanism by which miR-942 regulates ESCC stem cell-like traits through activating the Wnt/β-catenin pathway, suggesting that miR-942 might function as a therapeutic target for ESCC.

Esophageal cancer is one of the most aggressive cancers which ranks as the sixth cause of cancer-related deaths worldwide [28]. Esophageal squamous cell carcinoma (ESCC) occuring in the middle or upper third of the esophageal, and adenocarcinoma (EAC) occuring in the distant third or oesophgogastric junction, are the two major histologic subtypes of esophageal cancer. ESCC leads to significant morbidity and mortality in developing countries, such as China and Iran, where approximately 90% of all cases of esophageal cancer are ESCC [29]. Surgical resection combines with neoadjuvant chemo- or radio-therapy, are the common therapeutic methods used nowadays. However, despite advances in medical diagnosis and treatment, the prognosis of ESCC has not significantly improved, and the 5-year survival rate for ESCC patients ranges only between 15% and 40%. The most important reason results in poor prognosis of ESCC always due to delayed diagnosis, metastasis, highly relapse rate and a limited understanding of the cellular and molecular mechanisms underlying the initiation, promotion, and progression of ESCC. Nowadays, multiple researchers have proved that the existence of CSCs could lead to therapeautic failure of ESCC [30]. Therefore, targeting CSCs might develop be an effective strategy for cancer therapy, and it is important to explore the properities of CSCs [31, 32].

The Wnt/β-catenin signalling pathway is a well-known pathway involved in regulating self-renewal and oncogenesis in many systems [33, 34]. In normal physiological state, the Wnt/β-catenin signalling pathway regulates downstream genes involved in basic cellular processes, such as cell proliferation, differentiation, migration and cell death [35, 36]. It means that the Wnt/β-catenin signalling should be kept in a normal level to exert normal physiological function. However, extensive findings have shown that the Wnt/β-catenin pathway is constitutively activated in many cancers [37]. Understanding the underlying mechanisms is important in for cancer therapy. In the present study, we found miR-942 activates Wnt/β-catenin pathway in different layers through directly downregulation of sFRP4, GSK3β, and TLE1. Therefore, our results uncover a novel mechanism in which Wnt/β-catenin pathway constitutively activates in cancer.

sFRP4 is hommologous to the extracellular cysteine-rich domain of frizzled, which is a putative Wnt-binding receptor [38]. Through interaction and inhibition of Wnt ligand, sFRP4 exert antagonistic activity in Wnt/β-catenin pathway, suggesting that sFRP4 might be a tumour suppressor [39]. Consistently, sFRP4 has been found to be downregulated in several human cancers. Carmon et al. reported that sFRP4 was significantly decreased in endometrial cancer cells and inhibited cell growth [20]. Meanwhile, sFRP4 increases chemotherapeutic sensitivity in glioma stem-like cells and induces apoptosis via inhibiting Wnt/β-catenin signalling [40]. It has been reported that the protein expression of sFRP4 was downregulated through promoter CpG island methylation in colorectal cancer, mesothelioma, cervical cancer and leukemia [41-44]. However, Zinovyeva and his colleagues showed that the mRNA expression of sFRP4 was higher in ESCC than in normal cells [45], the molecular mechanism underlying sFRP4 protein downregulation in ESCC remained largely unclear. In the current study, we demonstrated first time that miR-942 directly targets the sFRP4-3′UTR, thereby inhibiting sFRP4 protein expression.

In summary, our studies demonstrated that overexpression of miR-942 prmotes stem cell-like traits and tumourigenesis in ESCC by directly suppressing sFRP4, GSK3β, and TLE1, which are multiple negative regulators of Wnt/β-catenin signalling. Our findings reveal a novel molecular mechanism to explain how constitutive activation of the Wnt/β-catenin pathway is maintained in cancers and suggests that miR-942 might serve as a potential therapeutic target for ESCC.

## MATERIALS and METHODS

### Cells

Primary cultures of normal esophageal epithelial cells (NEECs) were established from fresh specimens of the adjacent noncancerous esophageal tissue taken from an area over 5 cm from the cancerous tissue, according to previous report [46]. The esophageal cancer cell lines, including Kyse140, Kyse180, Kyse30, Kyse410, Kyse510, Kyse520, Eca109, TE-1, EC18, EC9706, HKESC1 and 108CA were grown in the DMEM medium (Invitrogen, Carlsbad, CA) supplemented with 10% fetal bovine serum (HyClone, Logan, UT).

### Patient information and tissue specimens

A total of 158 paraffin-embedded and archived ESCC samples, which were histopathologically and clinically diagnosed at the Department of Pathology, Affiliated Hospital of Academy of Military Medical Science from 2001 to 2006, were examined in this study. Prior patient consent and approval from the Institutional Research Ethics Committee were obtained for the use of these clinical materials for research purposes. Clinical information on the samples is summarized in Supplementary Table 1. Clinical pathological tumour-node-metastasis (TNM) staging was determined by the extent of tumour invasion in the esophageal wall and lymphatic and venous invasion status according to the criteria proposed by Union for International Cancer Control (UICC) criteria. Ten freshly collected ESCC tissues and the matched adjacent noncancerous esophageal tissues were frozen and stored in liquid nitrogen until further use.

### Primers and oligonucleotides

Cloning miR-942: 5′-GCCAGATCT TGATTGACTTACAGCCCAGTT-3′ and 5′-GCCGAA TTCCACCTGTCTTTATTCCACCC-3′; Cloning c-myc ORF: 5′-GCCAGATCTC TGGATTTT TTTCGGGTAGT-3′ and 5′-GCCGAATTCTTACGCACAAG AGTTCCGTA-3′; Cloning sFRP4 - 3′UTR-luci: 5′-GCCCCGCGGCTTGCCCTAACAACTCA-3′ and 5′-GCCCTGCAGTTTCCT TTGGGCGTTG-3′; Cloning GSK3β-3′UTR-luci: 5′-GCCCCGCGGATGTTTGCCGTGAGG A-3′ and 5′-GCCCTGCAGCCTGGGTCCACATATTTAC-3′; Cloning TLE1-3′UTR-luci: 5′-G CCCCGCGGCGTTTATAGTTGAATTGG-3′ and 5′-GCCCTGCAGTTATTGGTACTAAAGCC T-3′. For depletion of sFRP4, GSK3β, TLE1 and c-myc siRNA was synthesized and purified by RIBOBIO Company (Guangzhou, China).

### RNA extraction and real-time quantitative PCR (qRT-PCR)

Total cellular RNA was extracted using the TRIzol solution (Invitrogen), according to the manufacturer's protocol. Complementary DNAs were synthesized and Real-time PCR was performed using RT Real-Time^TM^ SYBR Green (Bio-Rad Laboratories, Berkeley, CA). The following primers were used: *ABCG2* forward: 5′-TGGTGTTTCCTTGTGACACTG-3′; *ABCG2* reverse: 5′-TGAGCCTTTGGTTAAGACCG-3′; *KLF4* forward: 5′-GTCA GTTCA TCTGAGCG GG-3′; *KLF4* reverse: 5′-AGAGTTCCCATCTCAAGGCA-3′; *SOX2* forward: 5′-GCTTAGCCTC GTCGATGAAC-3′; *SOX2* reverse: 5′-AACCCCAAGATGCACAACTC-3′; *OCT4* forward: 5′-G GTTCTCGATACTGGTTCGC-3′; *OCT4* reverse: 5′-GTGGAGGAAGCTGACAACAA-3; *NANOG* forward: 5′-ATGGAGGAGGGAAGAGGAGA-3′; *NANOG* reverse: 5′-GATTTGTGG GCCTGAAGAAA-3′; *sFRP4* forward: 5′-GTTCCTGCAGCCTCTCTTCC-3′; sFRP4 reverse: 5′-GTGTTACGAGTGGCGTTCAA-3′; *GSK3β* forward: 5′-ACTTCTTGTGGCCTGTCTGG-3′ *GSK3β* reverse: 5′-AGCTTTTGGCAGCATGAAAG-3′; *TLE1* forward: 5′-TCAATCTCTTGGC GATTTCA-3′ reverse: 5′-AAGACAGAAATGCAGAGGCA-3′; *GAPDH* forward: 5′-AATGAA GGGG TCATTGATGG-3′; *GAPDH* reverse: 5′-AAGGTGAAGGTCGGAGTCAA-3′. The housekeeping gene *GAPDH* and the small nuclear RNA U6 were used as internal controls for mRNAs and microRNA, respectively. Expression levels of genes were calculated as 2^−[(Ct of p21, p27, *Cyclin D1*) − (Ct of *GAPDH*)]^. The relative expression of the miRNA was calculated as 2^−[(Ct of miR-942) − (Ct of U6)]^. (C_t_ represents the threshold cycle for each transcript).

### Vectors, retroviral infection and transfection

The human miR-942 gene was PCR-amplified from genomic DNA and cloned into a pMSCV- puro retroviral vector. pMSCV-miR-942 was cotransfected with the pIK packaging plasmid in HEK293T cells using the standard calcium phosphate transfection method. Thirty-six hours after the cotransfection, supernatants were collected and incubated with cells to be infected for 24 hours in the presence of polybrene (2.5 μg/ml). After infection, puromycin (0.5 μg/ml) was used to select stably transduced cells over a 10-day period [47]. The reporter plasmids containing wild-type (CCTTTGATC; TOP flash) or mutated (CCTTTGGCC; FOP flash) TCF/LEF DNA binding sites were purchased from Upstate Biotechnology (New York, USA). The 3′UTRs of sFRP4, GSK3β, and TLE1, respectively, were amplified and cloned downstream to the luciferase gene in a modified pGL3 control vector. Antagomir-942 was purchased from RIBOBIO Company (Guangzhou, China).

### Luciferase reporter assay

Cells (3× 10^4)^ were seeded in triplicate in 24-well plates. 24-hours later, indicated luciferase reporter plasmids plus 3 ng pRL-TK Renilla plasmid were transfected into the cells using Lipofectamine 2000 Reagent (Life Technologies, USA). 48 hours after transfection, Dual Luciferase Reporter Assay (Promega, USA) was performed according to the manufacturer's instructions.

### Western blot analysis

Western blot analysis was performed using anti- sFRP4 (1:2000, Abcam, Cambridge, MA, USA); anti-GSK3β (1:5000, Abcam, Cambridge, MA, USA), anti-TLE1 (1:3000, Abcam, Cambridge, MA, USA); anti-β-catenin (1:5000, Abcam, Cambridge, MA, USA); anti-c-myc (1:1000, Abcam, Cambridge, MA, USA). To control sample loading, the blotting membranes were stripped and re-probed with an anti–α-tubulin or anti-p84 antibody (1:5000, Sigma, Saint Louis, MO, USA). Nuclear extracts were prepared using the Nuclear Extraction Kit (Active Motif), according to the manufacturer's instructions.

### miRNP immunoprecipitation

Cells were co-transfected with HA-Ago1 together with 100 nM miR-942, followed by HA-Ago1 immunoprecipitation using HA-antibody. Real-time PCR analysis of the IP material was used to test the association of the mRNA of sFRP4, GSK3β, and TLE1 with the RISC complex.

### Animal studies

BALB/c-nu mice (4–5 weeks of age, 18-20g) were purchased from the Center of Experimental Animal of Guangzhou University of Chinese Medicine. All experimental procedures were approved by the Institutional Animal Care and Use Committee of affiliated hospital of academy of military medical sciences. The BALB/c nude mice were randomly divided into two groups (*n=*6/group) and was inoculated subcutaneously with Eca109/Vector cells (1 × 10^6^) in the left dorsal flank and with Eca109/miR-942 cells (1 × 10^6^) in the right dorsal flank per mouse. For *in vivo* antagomir assay, 1×10^6^ Eca109 cells were inoculated subcutaneously in the left dorsal flank of BALB/c nude mice. 7 days later, one hundred microliters of antagomir −942 (diluted in PBS at 2 mg/ml) or control antagomir was administrated intratumourally 3 times per week for 2 weeks, starting when the average volume of grown tumours reached approximately 50 mm^3^. For dosages injection assay, the NOD/SCID mice were randomly divided into 3 groups (n= 6 per group). Indicated cells of 3 dosages (1 × 10^4^, 1 × 10^3^, and 1 × 10^2^) were inoculated subcutaneously with matrigel (final concentration of 25%) into the inguinal folds of NOD/SCID mice. Tumour volume was calculated using the equation (Length*Weight^2^)/2. On day 40, tumours were detected by an IVIS imaging system (Caliper), then animals were euthanized, and the tumours were excised, weighed and paraffin-embedded.

### Tumour sphere formation assays

Five hundred cells were seeded in 6-well ultra-low cluster plates and 10 or 20 cells were seeded in 24-well ultra-low cluster plates for 10 days. Spheres were cultured in DMEM/F12 serum-free medium (Invitrogen, USA) supplemented with 2% B27 (Invitrogen, USA), 20 ng/ml EGF, 20 ng/ml bFGF, and 5 μg/ml insulin (PeproTech, USA).

### Flow cytometric analysis

Flow cytometric analysis or flow cytometric cell sorting was conducted using phycoerythrin (PE)-conjugated monoclonal mouse anti-human CD90 (Miltenyi Biotec GmbH, Germany). Samples were analyzed and sorted on BD FACS Canto II and FACS Aria I, respectively (BD Biosciences, USA) with data analyzed using FlowJo software (Tree Star Inc, USA).

### Chromatin Immunoprecipitation (ChIP)

Cells (2×10^6^) in a 100-mm culture dish were treated with 1% formaldehyde to cross-link proteins to DNA. The cell lysates were sonicated to shear DNA to sizes of 300-1000 bp. Equal aliquots of chromatin supernatants were incubated with 1 μg of anti-c-myc antibody (Abcam, Cambridge, MA) or an anti-IgG antibody (Millipore, Billerica, MA) overnight at 4°C with rotation. After reverse cross-link of protein/DNA complexes to free DNA, PCR was performed.

### Bioinformatics analysis

The following on-line software programs were used for bioinformatics analysis: The Cancer Genome Atlas (TCGA) (http://cancergenome.nih.gov/); TargetScan 6.2 (http://www.targetscan.org/); and miRanda (http://www.microrna.org/microrna/getGeneForm.do).

### Statistical analysis

All statistical analyses were carried out using SPSS 18.0 statistical software. The Kaplan-Meier method was used to establish survival curves, and the survival differences were compared using the log-rank test. Continuous data were compared using Student's 2-tailed t-test. Multivariate statistical analysis was performed using a Cox regression model. In all cases, *P* < 0.05 was considered statistically significant.

### Study approval

For the use of materials for research purposes, prior patient consent was obtained, and study approval was granted by the affiliated hospital of academy of military medical sciences and Cancer Center Institution Board. All animal studies were approved by the Affiliated hospital of Academy of military medical sciences Institutional Animal Care and Use Committee.

## SUPPLEMENTARY MATERIAL, FIGURES AND TABLES


