# Supplementary material for: miR-942 promotes cancer stem cell-like traits in esophageal squamous cell carcinoma through activation of Wnt/β-catenin signalling pathway
Source: Oncotarget. 2015 Mar 30;6(13):10964–77. doi: 10.18632/oncotarget.3696 (PMC4484432; doi:10.18632/oncotarget.3696)
Supplement: Supplementary file 1 [file oncotarget-06-10964-s001.pdf]

miR-942 promotes cancer stem cell-like traits in esophageal squamous cell carcinoma through activation of Wnt/β-catenin signalling pathway

Supplementary Material

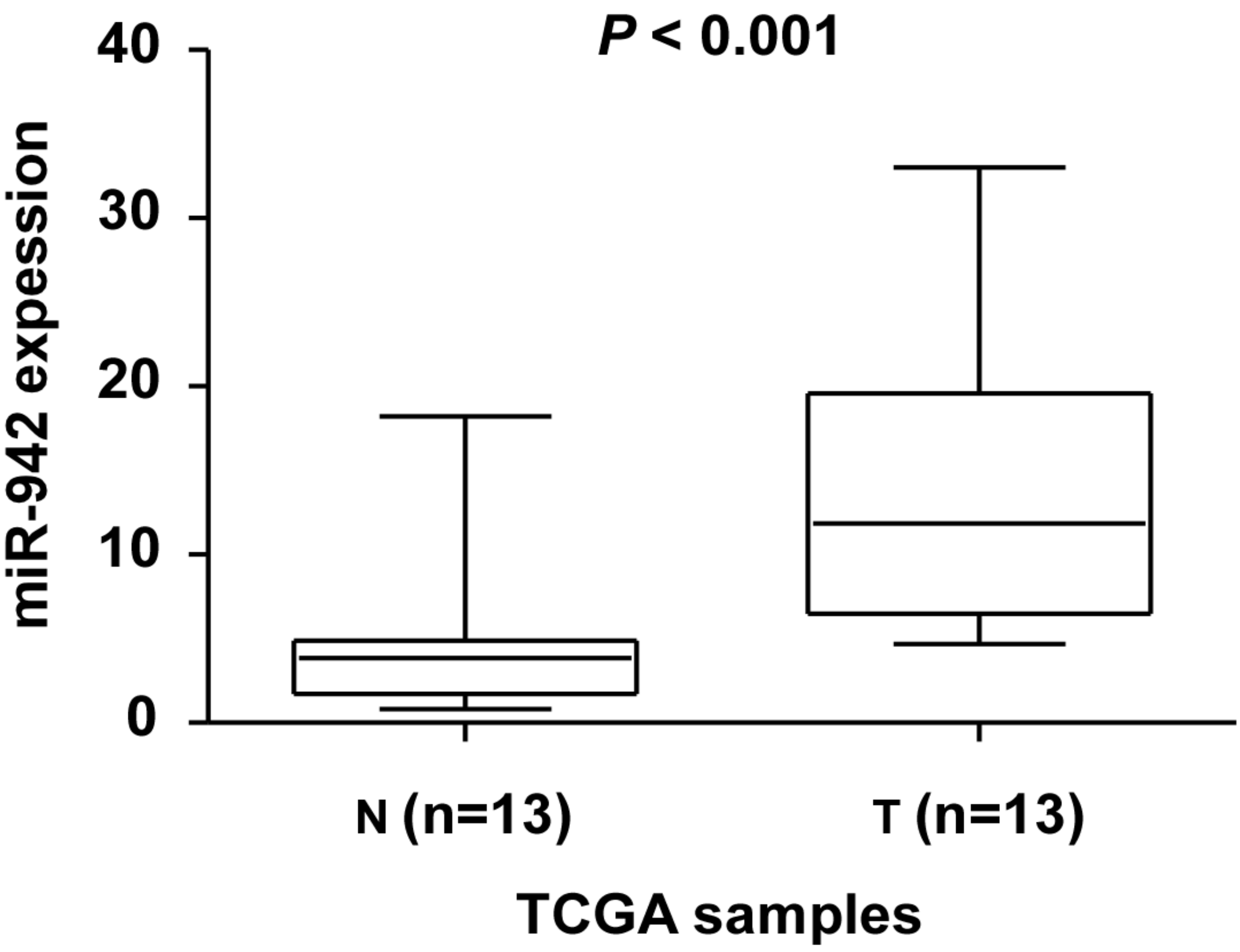

Supplementary Figure 1. Expression profiling of miR-942 from the Cancer Genome Atlas (TCGA) datasets in paired primary esophageal tumors (T) and the adjacent normal tissues (ANT) (n = 13; *P< 0.001)*.

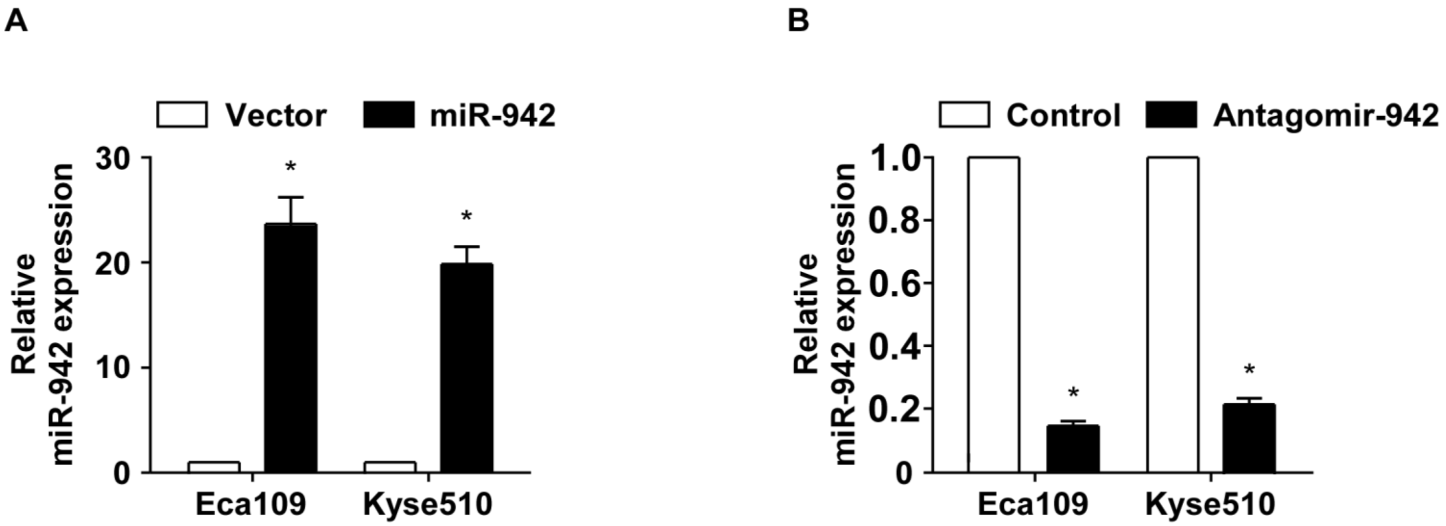

Supplementary Figure 2. Relative miR-942 expression in miR-942-overexpressing (A) or miR-942-silenced cells (B). Each bar represents the mean ± SD of three independent experiments. **P* < 0.05.

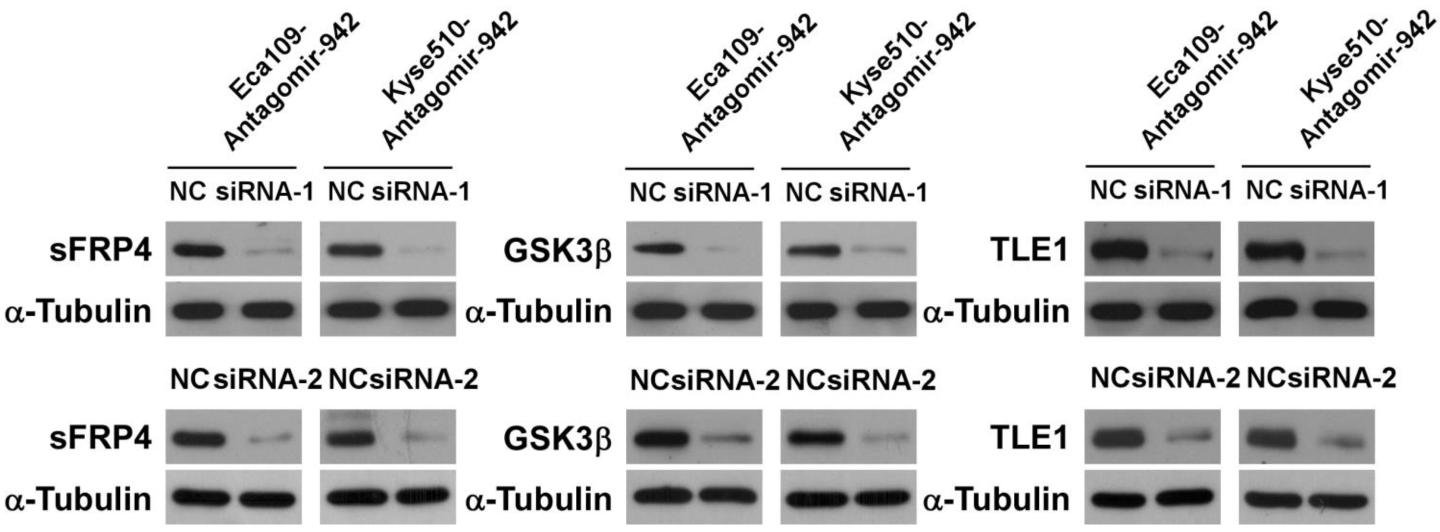

Supplementary Figure 3. Western blot analysis of the expression levels of sFRP4, GSK3β, and CtBP2 expression in antagomir-942 cells which transfected with sFRP4-siRNA (left panel), GSK3β-siRNA (middle panel), or CtBP2-siRNA (right panel). α-Tubulin served as the loading control.

Supplementary Table 1. Clinicopathological characteristics and expression of miR-942 in studied ESCC patients

Factor	No.	(%)
**Gender**		
Male	116	73.4
Female	42	26.6
**Age (years)**		
≤57	89	56.3
>57	69	43.7
**Clinical stage**		
I	20	12.7
II	69	43.7
III	53	33.5
IV	16	10.1
**T classification**		
T_1	18	11.3
T_2	49	31.0
T_3	83	52.5
T_4	8	5.0
**N classification**		
N_0	86	54.4
N_1	72	45.6
**M classification**		
No	144	91.1
Yes	14	8.9
**Histological differentiation**		
Well	47	29.7
Moderate	66	42.0
Poor	45	28.3
**Vital status**		
Alive	54	34.2

Dead	104	65.8
**Expression of miR-942**		
Low expression	79	50.0
High expression	79	50.0

Supplementary Table 2. Correlation between the clinicopathological features and expression of miR-942

Patient characteristics		miR-942 expression		*P*-value
		Low	High	
**Gender**	Male	57	59	0.552
	Female	22	20	
**Age (years)**	≤57	49	50	0.612
	>57	30	29	
**Clinical stage**	I	14	6	< 0.001
	II	30	39	
	III	16	37	
	IV	5	11	
**T classification**	T_1	10	8	0.005
	T_2	22	27	
	T_3	34	49	
	T_4	3	5	
**N classification**	N_0	51	35	< 0.001
	N_1	23	49	
**M classification**	No	70	74	0.004
	Yes	4	10	
**Histological differentiation**	Well	27	20	0.045
	Moderate	24	42	
	Poor	16	29	
**Vital status**	Alive	30	24	< 0.001
	Dead	35	69	

Supplementary Table 3. Univariate and multivariate analysis of different prognostic parameters in patients with ESCC by Cox-regression analysis

	Univariate analysis		Multivariate analysis	
	P	**Hazard ratio (95% CI)**	***P***	**Hazard ratio (95% CI)**
**Clinical stage**				
I<br>II<br>III<br>IV	<0.001	1.985 (1.435-2.529)	0.312	1.314 (0.953-2.043)
**miR-942 expression**				
Low expression<br>High expression	<0.001	2.931 (2.223-4.012)	<0.001	2.352 (1.820-3.380)
